# Physiological extremes of the human blood metabolome: A metabolomics analysis of highly glycolytic, oxidative, and anabolic athletes

**DOI:** 10.14814/phy2.14885

**Published:** 2021-06-21

**Authors:** Daniela Schranner, Martin Schönfelder, Werner Römisch‐Margl, Johannes Scherr, Jürgen Schlegel, Otto Zelger, Annett Riermeier, Stephanie Kaps, Cornelia Prehn, Jerzy Adamski, Quirin Söhnlein, Fabian Stöcker, Florian Kreuzpointner, Martin Halle, Gabi Kastenmüller, Henning Wackerhage

**Affiliations:** ^1^ Exercise Biology Department of Sport and Health Sciences Technische Universität München Munich Germany; ^2^ Institute of Computational Biology Helmholtz Zentrum München Neuherberg Germany; ^3^ University Center for Prevention and Sports Medicine University Hospital Balgrist Universität Zürich Zurich Switzerland; ^4^ Department of Neuropathology Institute of Pathology Technische Universität München Munich Germany; ^5^ Department of Prevention and Sports Medicine Technische Universität München Munich Germany; ^6^ Research Unit Molecular Endocrinology and Metabolism Helmholtz Zentrum München Neuherberg Germany; ^7^ German Center for Diabetes Research Neuherberg Germany; ^8^ Chair of Experimental Genetics Technische Universität München Freising‐Weihenstephan Germany; ^9^ Department of Biochemistry Yong Loo Lin School of Medicine National University of Singapore Singapore; ^10^ Teaching and Educational Lab Department of Sport and Health Sciences Technische Universität München Munich Germany; ^11^ Prevention Center Department of Sport and Health Sciences Technische Universität München Munich Germany

**Keywords:** athlete, energy metabolism, exercise biomarker, exercise phenotype

## Abstract

Human metabolism is highly variable. At one end of the spectrum, defects of enzymes, transporters, and metabolic regulation result in metabolic diseases such as diabetes mellitus or inborn errors of metabolism. At the other end of the spectrum, favorable genetics and years of training combine to result in physiologically extreme forms of metabolism in athletes. Here, we investigated how the highly glycolytic metabolism of sprinters, highly oxidative metabolism of endurance athletes, and highly anabolic metabolism of natural bodybuilders affect their serum metabolome at rest and after a bout of exercise to exhaustion. We used targeted mass spectrometry‐based metabolomics to measure the serum concentrations of 151 metabolites and 43 metabolite ratios or sums in 15 competitive male athletes (6 endurance athletes, 5 sprinters, and 4 natural bodybuilders) and 4 untrained control subjects at fasted rest and 5 minutes after a maximum graded bicycle test to exhaustion. The analysis of all 194 metabolite concentrations, ratios and sums revealed that natural bodybuilders and endurance athletes had overall different metabolite profiles, whereas sprinters and untrained controls were more similar. Specifically, natural bodybuilders had 1.5 to 1.8‐fold higher concentrations of specific phosphatidylcholines and lower levels of branched chain amino acids than all other subjects. Endurance athletes had 1.4‐fold higher levels of a metabolite ratio showing the activity of carnitine‐palmitoyl‐transferase I and 1.4‐fold lower levels of various alkyl‐acyl‐phosphatidylcholines. When we compared the effect of exercise between groups, endurance athletes showed 1.3‐fold higher increases of hexose and of tetradecenoylcarnitine (C14:1). In summary, physiologically extreme metabolic capacities of endurance athletes and natural bodybuilders are associated with unique blood metabolite concentrations, ratios, and sums at rest and after exercise. Our results suggest that long‐term specific training, along with genetics and other athlete‐specific factors systematically change metabolite concentrations at rest and after exercise.

## Graphical abstract

Years of training and favorable genetics combine to physiologically extrem forms of metabolism in competitive athletes. We compared the serum metabolomes of sprinters, natural bodybuilders, endurance athletes and untrained subjects. Natural bodybuilders and endurance athletes have unique metabolomes after an overnight fast and after fasted maximum exercise whereas sprinters are more similar to untrained controls.

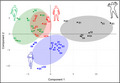

## 1 INTRODUCTION

Inactivity, overweight and its negative impacts on health are a world‐wide problem (Afshin et al., 2017). In contrast, physical activity and exercise training are widely accepted as health‐promoting factors (Afshin et al., 2017; Blair, 2009; Cohen et al., 2015; Pedersen & Saltin, 2015). Therefore, a major goal in exercise science is to understand how exercise triggers physiological adaptation (e.g., an increase in muscle mass or aerobic capacity) and how these adaptations can benefit health or mitigate disease (Pedersen & Saltin, 2015).

In athletes, years of training plus a unique genetic makeup (Sarzynski & Bouchard, 2020) result in metabolic adaptations: Endurance training increases mitochondrial content and activity of oxidative enzymes in skeletal muscle (Egan & Zierath, 2013), resistance training increases muscle fiber size (Mero et al., 2013), and anerobic training like sprint training increases glycolytic enzymes in skeletal muscle (Ross & Leveritt, 2001). Eventually, genetics and years of specific training in athletes result in physiologically extreme metabolic phenotypes.

Metabolites can serve as molecular read‐outs of these metabolic phenotypes (Aebersold & Mann, 2016; Patti et al., 2012). The metabolome, which comprises all metabolites within an organism, is highly dynamic, and susceptible to external influences like exercise (Krug et al., 2012). Studies have shown that one bout of exercise (Contrepois et al., 2020; Morville et al., 2020; Schranner et al., 2020) and exercise training for several weeks (Felder et al., 2017; Neal et al., 2013) change hundreds of metabolites in blood.

A targeted change of specific metabolites through exercise could be directly relevant to diseases with dysregulated metabolism. Recently, Morville et al. showed that a short term, targeted change of metabolites is possible through different exercise modes. They showed that within one session, endurance exercise changes different metabolites than resistance exercise does (Morville et al., 2020). However, it is not clear if there is a long‐term effect of different exercise modes on the metabolome.

While short‐term metabolite changes after one exercise session in athletes were reported (Al‐Khelaifi et al., 2018, 2019; Breit et al., 2015; Coelho et al., 2016; Hall et al., 2016; Howe et al., 2018), no study showed how years of metabolic adaptation to endurance, resistance, or sprint training affect metabolite changes to the same acute exercise.

Therefore, we wanted to find out how long‐term physiological adaptation to different exercise training modes (endurance, resistance, sprint) affect the metabolome at rest and how these different adaptations affect the metabolic response to the same acute exercise. By comparing the serum metabolomes of glycolytic sprinters, oxidative endurance athletes, and anabolic natural bodybuilders before and after a maximum graded exercise test, we aimed to answer the following research questions:

1) Do sprinters, endurance athletes and natural bodybuilders have distinct blood metabolite concentrations? If so, the concentrations of which metabolites explain the differences in‐between athlete groups?

2) Within these highly trained athletes, how does a bout of graded cycle exercise to exhaustion affect blood metabolite concentrations? And specifically, do metabolite concentrations change differently after the exercise depending on the athlete group?

## 2 MATERIALS AND METHODS

### 2.1 Study cohort and human exercise testing

For this study, we recruited three groups of healthy male athletes (*n* = 15): 5 sprinters, 6 endurance athletes, 4 natural bodybuilders, and 4 healthy untrained males. All participants passed the inclusion criteria (Supplementary Table S1) and completed the study. Mean group characteristics (Table 1) and individual details (Supplementary Table S2) are provided. In preparation for the study, participants followed a standard diet (Supplementary Table S7) on the day before testing, refrained from exercise training for 24 hours and from dietary supplements for 48 hours before testing. Participants recorded their exercise training for 4 weeks (Table 1) and their intake of dietary supplements and medication for one week before the study (Supplementary Table S2).

**TABLE 1:** Mean (SD) group characteristics of study participants showing significantly different groups (*p* < 0.05) in bold after correcting for multiple testing

	Control	Natural Bodybuilding^a^	Endurance	Sprint
Number of subjects	4	4	6	5
Age (years)	30 (2)	28 (6)	30 (3)	24 (3)
Resting heart rate (bpm)	70 (9)	56 (4)	51 (15)	59 (7)
Resting blood pressure (mmHg)	140/80 (12/7)	123/78 (10/4)	122/75 (11/7)	126/78 (9/9)
BMI (kg/m^2^)	24.8 (2.3)	**26**.**5 (2.7)** ^b^	22.1 (1.9)	21.9 (1.5)
Height (cm)	188 (4.5)	172 (6.6)	183 (3.8)	189 (7.0)
Body fat (%)	18.8 (7.7)	10.6 (1.2)	**7.5 (1.0)** ^c^	**5.5. (0.7)** ^c^
Upper arm circumference (cm)	30.1 (2.7)	33.1 (3.7)	28.1 (1.7)	28.3 (1.8)
Thigh circumference (cm)	54.2 (4.2)	59.5 (6.6)	51.4 (5.1)	55.4 (4.1)
Subcutaneous fat upper arm (mm)	**14**.**4 (7.2)** ^b^, ^d^, ^e^	6.4 (0.5)	6.5 (2.1)	5.6 (0.9)
Subcutaneous fat thigh (mm)	**20**.**4 (7.3)** ^b^, ^d^, ^e^	10.1 (2.7)	7.7 (3.4)	5.8 (1.7)
Ventilatory threshold 1 (ml/kg^/^min)	26.2 (2.1)	27.2 (3.0)	**47.9 (6.0)** ^b^, ^c^, ^d^	27.3 (5.6)
VO_2_max (ml/kg/min)	45.6 (4.7)	41.8 (2.0)	**63.6 (6.6)** ^b^, ^c^, ^d^	52.6 (5.4)
Relative maximum workload (W/kg)	3.9 (0.5)	3.7 (0.2)	**5.9 (0.3)** ^b^, ^c^, ^d^	**4.8 (0.2)** ^c^, ^d^
Lactate (mmol/l) 4 min after maximum workload	12.0 (1.0)	9.7 (0.9)	9.5 (2.6)	13.0 (1.5)
Lactate (mmol/l) 10 min after maximum workload	12.1 (1.7)	9.2 (2.7)	**5.9 (1.7)** ^b^, ^c^	11.4 (3.1)
Reactive strength (RSI)	111 (30)	125 (25)	164 (29)	**218 (37)** ^c^, ^d^
Hand grip strength (kg)	59.3 (7.2)	62.4 (6.6)	51.5 (2.8)	60.2 (5.1)
Endurance training (min/week)	41 (63)	80 (40)	**815 (317)** ^b^, ^c^, ^d^	162 (65)
Resistance training (min/week)	0 (0)	**413 (227)** ^c^, ^e^	85 (50)	207 (56)
Speed training (min/week)	0 (0)	135 (201)	65 (84)	**294 (161)** ^c^

^a^ Natural bodybuilders are bodybuilders who abstain from performance enhancing drugs listed in the World Natural Bodybuilding Federation banned substances list e.g. steroid hormones (Liokaftos, 2018).

^b^ significantly different from sprinters.

^c^ significantly different from controls.

^d^ significantly different from natural bodybuilders.

^e^ significantly different from endurance athletes.

**FIGURE 1:**
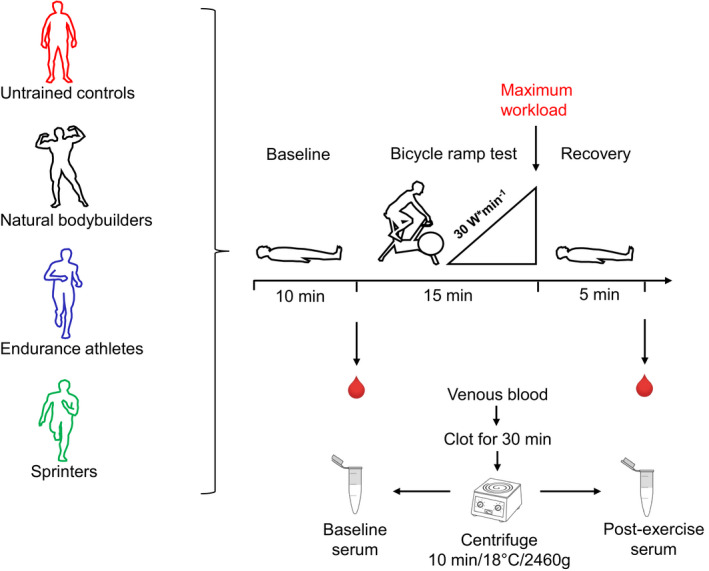
Overview of the study design where a standardized bicycle ramp test was performed with a continuously increasing load of 30 watts per minute until voluntary exhaustion

Human exercise testing included three phases: baseline measurement, exercise testing, and recovery (Figure 1). To reduce circadian bias, all participants reported to the laboratory at 7 am after a 10 hour overnight fast. Upon arrival, we measured height, weight, body circumferences, and body fat including measurement of subcutaneous fat over Biceps brachii and Quadriceps femoris. Body fat was calculated from the thickness of seven skin folds (7‐point‐calipermetry) using the method by Jackson & Pollock (Jackson & Pollock, 1978). After resting for 10 minutes, we took blood samples from an antecubital vein of the right arm in a supine position. After a three‐minute warm up, subjects performed a ramp‐test on a bicycle ergometer (Lode, Groningen, Netherlands) with power increasing linearly at a rate of 30 W per minute until voluntary exhaustion. During cycling, we continuously measured gas exchange with a stationary cardiopulmonary exercise testing system (Cortex, Germany). Out of 19 subjects, 18 met objective exhaustion criteria of either a respiratory exchange ratio (RER) >1.0 or a ventilatory equivalent of oxygen (VEeqO_2_) of >30.0 (Aspenes et al., 2011). One endurance athlete, E1 did not meet these criteria. Despite endurance trained subjects have lower RER than non‐endurance trained subjects in response to similar relative exercise intensity (Jeukendrup et al., 1997) we conclude subject E1 was not entirely physically exhausted. Five minutes after the end of exercise, we took a second blood sample from the antecubital vein of the left arm in a supine position. At maximum exhaustion, we started to sample lactate from the earlobe in 20 µl capillaries (EKF diagnostics, Germany) every 2 min for 10 min in total and analyzed samples immediately (Biosen S‐Line Analyzer, EKF diagnostics, Germany). Then participants rested for 90 minutes and ingested drinks and foods ad libitum. After rest, participants re‐warmed for ~15 minutes (10 min ergometry at 100 W and 5 min supervised jumping and dynamic stretching exercises). After re‐warm, reactive strength was measured by a drop jump from 30 cm height with a force plate (Kistler GmbH, Germany). The best out of three attempts (highest RSI) was recorded. Afterwards, we measured maximum hand grip force with a hand grip dynamometer (Jamar, JLW instruments, USA) where the best out of three attempts was recorded as well.

### 2.2 Blood sample preparation

We drew venous blood samples in four replicates into 9 ml serum S‐Monovettes Z‐Gel collecting tubes (Sarstedt AG und Co KG, Nuembrecht, Germany) at each timepoint. Clotting was allowed at room temperature for 30 min in an upright position. After centrifugation (10 min / 18°C, 2460 g), we merged the serum replicates into one 15 ml Falcon tube (Greiner Bio‐One GmbH, Kremsmuenster, Austria). Then, we aliquoted the serum into cryotubes (Sarstedt AG und Co KG, Nümbrecht, Germany), froze aliquots on dry ice for ~30 min and stored them at −80°C until analysis.

### 2.3 Metabolomics measurement

Blood serum samples were analyzed at the Genome Analysis Center at the Helmholtz Zentrum München (Munich, Germany) with a kit‐based metabolomics approach (Absolute*IDQ* p180 Kit; Biocrates Life Sciences AG, Innsbruck, Austria) applying liquid chromatography (LC‐MS/MS) and flow injection analysis‐tandem mass spectrometry (FIA‐MS/MS) to measure a pre‐defined set of 188 metabolites in a targeted fashion. Sample preparation and MS/MS measurements were performed according to the manufacturer's instructions (manual UM‐P180) as described previously (Zukunft et al., 2013). Briefly, 10 µL blood serum were placed into the 96‐well plate of the p180‐kit and dried in a nitrogen stream for 30 minutes. For tagging amino acids and biogenic amines, samples were derivatized with an excess of 5% phenylisothiocyanate (Sigma‐Aldrich, Steinheim, Germany). After drying under nitrogen, metabolites were extracted in 300 µL methanol (AppliChem, Darmstadt, Germany) containing 5 mM ammonium acetate (Sigma‐Aldrich, Steinheim, Germany). After incubation for 30 min at room temperature and centrifugation, the eluate was split and diluted for the following MS/MS analyses. For sample preparation and MS/MS analysis, we used the following laboratory equipment: (i) Hamilton Microlab STAR^TM^ robot (Hamilton Bonaduz AG, Bonaduz, Switzerland) for liquid sample handling, (ii) Ultravap nitrogen evaporator (Porvair Sciences, Leatherhead, UK) for sample drying, (iii) 1200 Series HPLC (Agilent Technologies Deutschland GmbH, Böblingen, Germany) equipped with a HTC PAL auto sampler (CTC Analytics, Zwingen, Switzerland) for the liquid chromatography step, and (iv) API 4000 triple quadrupole (Sciex Deutschland GmbH, Darmstadt, Germany) operated using the software Analyst (version 1.6.2) for MS/MS analysis. For compound identification and quantification, the mass spectrometer was run in multiple reaction monitoring mode. Following the kit procedure, we applied the MultiQuant 3.0.1 (Sciex) and Met*IDQ*™ software to assess measurement quality and to calculate metabolite concentrations in reference to the corresponding isotope‐labeled internal standards contained in the kit plate. Concentrations were reported in µM.

The assay allows simultaneous quantification of 188 metabolites: free carnitine (C0), 39 acylcarnitines (Cx:y), 21 amino acids (19 proteinogenic +citrulline + ornithine), 21 biogenic amines, hexose (sum, consisting of about 90%–95% glucose), 90 glycerophospholipids 14 lysophosphatidylcholines (lysoPC) and 76 phosphatidylcholines and 15 sphingolipids (SMx:y). The abbreviations Cx:y are used to describe the total number of carbons and double bonds of all fatty acid chains, respectively. PCs are labeled as either diacyl‐phosphatidylcholines (PC aa) or alkyl‐acyl‐phosphatidylcholines (PC ae). This labeling is based on the assumption that even‐numbered fatty acids and lower degrees of desaturation are more common than odd‐numbered fatty acids or very high degrees of desaturation. For example, the labels PC ae C38:0, PC aa C37:0, and PC aa C38:7, all have the same mass and, thus, all would describe the same PC kit measure representing a mixture of these structurally different PCs; according to the assumptions above, the respective kit measure is labeled as PC ae C38:0 (for more details see (Quell et al., 2019)).

The values for limit of detection (LODs) of metabolites were calculated as three times the values of the zero samples, here phosphate‐buffered saline. To assess the experimental variation of measurements, five aliquots of a pooled reference plasma (Haid et al., 2018) were measured on the same kit plate as the samples of interest and were used to calculate the coefficient of variation (CV) for each metabolite.

For a full list of all measured 188 metabolites and 44 calculated biologically relevant metabolite ratios or sums see Supplementary Table S3.

### 2.4 Statistical analysis

#### 2.4.1 Data quality control and preprocessing

In total, 37 metabolites and 1 ratio were excluded from further analysis based on the following criteria: (i) missing values for more than 90% of the samples (c4‐OH‐Pro, Dopamine, Nitro‐Tyr, Carnosine), (ii) CV of the five reference plasma samples (indicating the technical variation of measurements) exceeding 25% (Zhang et al., 2020) (DOPA, Histamine, PC aa C30:2, SM C22:3, PEA, Spermine, SM C26:0, Spermine/Spermidine, C5:1‐DC), and (iii) values below specified LOD for more than 50% of samples (29 metabolites, including DOPA, PEA, Spermine, C5:1‐DC). Parameters for quality control (missingness, CV, LOD) along with the mean concentrations of all measured analytes are provided in Supplementary Table S3.

As metabolite concentrations are mostly log‐normally distributed, the 151 metabolites and 43 ratios remaining after quality control were log‐transformed (log2) and missing values (PC ae C30:1, 5.3%; PC ae C38:1, 18.4%) were imputed using a k‐nearest‐neighbor approach (k=3) with variable selection (Do et al., 2018).

#### 2.4.2 Partial least squares discriminant analysis (PLS‐DA)

To check if the four groups (control, endurance, sprint, bodybuilding) can be discriminated based on their metabolomic profiles at baseline and after exercise, we performed a PLS‐DA analysis using the ropls R package (version 1.16.0). Prior to the analysis, metabolite concentrations were scaled (mean = 0, standard deviation = 1) over all samples. PLS‐DA projects these z‐scores of the 194 metabolite measures onto a reduced number of artificial components (here: components 1 and 2) that are linear combinations of the original variables which maximize the distinction of the pre‐defined groups (i.e., the covariance between components built from the variables and response (grouping)) (Wold et al., 2001). R2X (0.258), R2Y (0.488), Q2Y (0.304) representing the fraction of explained variances (of variables and response) and the accuracy of prediction (Eriksson, 2002; Tenenhaus, 1998) are provided as measures for the quality of the resulting PLS‐DA model in Supplementary Figure S1. To test for overfitting, we inspected R2Y and Q2Y from 20 PLS‐DA models based on our data with random permutations of the group labels. Resulting empiric *p*‐values for the achieved R2Y and Q2Y were below 0.05 (Szymańska et al., 2012). To identify key variables that drive the discrimination of athlete groups, we examined the loading vectors of the two PLS‐DA components (i.e., the weights assigned to each metabolite in the linear combination that defines each component). The loadings along with the metabolites' variable importance in projection (VIP) scores (summarizing the loading weights for both components and how much the components explain the group distinctions) are given in Supplementary Table S4 and Supplementary Figure S1. To examine the influence of sums and ratios (as partially redundant variables) on the separation of groups in the PLS‐DA, we repeated the analysis based on the 151 single metabolites and found the group differences to be stable (Supplementary Figure S2).

#### 2.4.3 Hypothesis testing for group differences in metabolite levels

For the metabolites that showed the five most extreme loading weights in negative and positive direction in the two PLS‐DA components, we formulated a linear mixed effects model (assuming fixed effects for time (baseline/post exercise) and group and a random intercept for the subjects) and performed an ANOVA to obtain *p*‐values for the group differences in the levels of these 2*10 selected metabolites (z‐scored). Group effects were considered significant if *p* < 0.0050 (α = 0.05/10 adjusted for ten tests). As component 1 separates the bodybuilding group and component 2 the endurance group from the other groups, we additionally tested for differences of these two groups against all other participants for the selected metabolites respectively, again using analogous linear mixed models.

#### 2.4.4 Hypothesis testing for effect of exercise and effect differences by group

To identify metabolites or ratios/sums that significantly change upon exercise, we performed a paired Student's t‐test for each of the 194 variables. The resulting ‐log10(*p*‐value) were displayed versus the means of metabolite fold changes within individuals of measured metabolite concentrations in a Volcano plot (generated using Prism 8.3.0, GraphPad). Log2 fold changes (means of fold change within individuals) and *p*‐values for the full list of metabolites are provided in Supplementary Table S5. *p*‐values were considered significant if *p* < 2.58*10^−4^ (α = 0.05/194 adjusted (Bonferroni) for multiple testing). For identification of group‐specific effects of exercise, we performed a *t*‐test for each metabolite comparing the mean log2 fold change (within individuals) of each group with the mean log2 fold change (within individuals) of all other subjects (Supplementary Table S6). Additionally, we performed non‐parametric Wilcoxon signed rank/rank tests to ensure robustness of our results against potential outliers (Supplementary Tables S5 and S6).

All calculations were performed using R Studio (Version 1.2.5033, Boston, MA, USA) with R version 3.6.2. Single metabolite plots, the PLS‐DA loading plot and the Volcano plot were generated using Prism version 8.3.0.

## 3 RESULTS

We recruited three groups of healthy male athletes: 5 sprinters, 6 endurance athletes, and 4 natural bodybuilders. Four healthy sedentary males were recruited as a control group. All subjects met the inclusion criteria given in Supplementary Table S1 and completed the study. Mean group characteristics are listed in Table 1 and subjects’ details are provided in Supplementary Table S2.

We analyzed 194 blood metabolite measures before and after the graded bicycle test to exhaustion to identify differences in the metabolomes of our four subject groups (Figure 1). These measures include 151 metabolites and 43 biologically relevant metabolite ratios and sum that remained after quality control of the analytical data (see Methods). We used a targeted metabolomics kit that mainly measures amino acids and lipids. Measured lipids include acylcarnitines, which are essential for fat metabolism and complex lipids such as sphingomyelins (SMs), and phosphatidylcholines (PCs), which are incorporated into membranes and carry two fatty acid residues. The kit also includes lysophosphatidylcholines (lysoPCs), which are degradation products of PCs. Over 70 PCs with different molecular weights are measured by the kit and are labeled as either diacyl‐phosphatidylcholines (PC aa) or alkyl‐acyl‐phosphatidylcholines (PC ae), also known as ether lipids. This labeling bases on the assumption that even‐numbered fatty acids and lower degrees of desaturation are more common than odd‐numbered fatty acids or very high degrees of desaturation. Common violations of these assumptions and observable mixed molecular compositions of measured PCs in human blood have been discussed recently in more detail (Quell et al., 2019). The assessed biologically relevant ratios include e.g. the carnitine‐palmitoyl‐transferase‐1 ratio (CPT1‐ratio), calculated as the concentration ratio of the CPT‐1 reaction products hexadecanoylcarnitine (C16:0) and octadecanoylcarnitine (C18:0) to the substrate free carnitine (C0). The CPT1‐ratio is considered a proxy measure of ß‐oxidation activity.

After quality control, the final set of 194 metabolite measures included 151 metabolites and 43 biologically relevant ratios or sums, which we used for all further statistical analyses (Supplementary Table S3).

### 3.1 Do sprinters, endurance athletes and natural bodybuilders have distinct blood metabolite concentrations at rest and after exercise?

**FIGURE 2:**
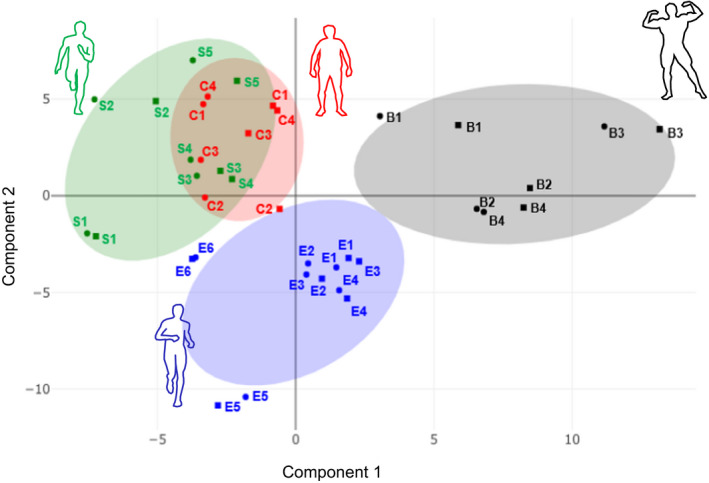
PLS‐DA score plot showing baseline (●) and post‐exercise (■) serum metabolite profiles within 75% confidence intervals (shading) of natural bodybuilders (B1‐B4), endurance athletes (E1‐E6), sprinters (S1–S5) and untrained controls (C1–C4)

We calculated a partial least squares discriminant analysis (PLS‐DA) by combining baseline and post‐exercise samples and using all 194 metabolite measures. PLS‐DA combines the large number of metabolite concentrations to yield two artificial components (component 1 and component 2) that are calculated to maximize the distance between the groups. Our PLS‐DA revealed overlapping clusters of controls and sprinters suggesting more similar metabolite concentrations. Natural bodybuilders (along component 1) and endurance athletes (along component 2) appeared as distinct clusters from sprinters and untrained controls (Figure 2) suggesting unique metabolite concentrations.

### 3.2 Which metabolites explain the differences in‐between groups?

In a next step, we identified those metabolites that separated the clusters of natural bodybuilders and those of endurance athletes from the other groups. We inspected the PLS‐DA loadings which show by how much each metabolite contributes to component 1 and component 2. We selected the five metabolites with the highest positive and negative contributions (i.e., largest absolute weights), respectively, for each component (Table 2). All 10 metabolites selected for component 1 (Table 2a) differed significantly in‐between groups in an ANOVA. Out of the 10 metabolites selected for component 2 (Table 2b), four differed significantly in‐between groups.

**TABLE 2:** Metabolites that contributed most to the distinction of the natural bodybuilders in component 1 (a) and to the distinction of endurance athletes in component 2 (b) from all other subjects

(a) Metabolite, ratio or sum	Loading on component 1^a^	*p* value Overall group differences	*p* value Natural Bodybuilder versus others
Isoleucine	*−0.1500*	2.7*10^−6*^	0.021
BCAA	*−0.1397*	1.1*10^−5*^	0.014
Leucine	*−0.1375*	6.5*10^−5*^	0.023
C14:2	*−0.1344*	2.3*10^−3*^	5.0*10^−3*^
Tryptophan	*−0.1269*	3.8*10^−6*^	0.011
PC ae C38:0^b^	0.1603	3.2*10^−7*^	6.8*10^−4*^
Total SM‐OH	0.1553	4.8*10^−5*^	0.014
SM (OH) C22:2^b^	0.1501	1.6*10^−4*^	0.0260
PC aa C36:6	0.1490	2.9*10^−7*^	2.2*10^−3*^
SM (OH) C22:1^b^	0.1488	3.5*10^−6*^	0.015

(b) Metabolite, ratio or sum	Loading on component 2^c^	*p* value Overall group differences	*p* value Endurance versus others
LysoPC a C18:2	*−0.1426*	0.018	0.138
Kynurenine/tryptophan	*−0.1411*	0.050	0.054
LysoPC a C18:1	*−0.1370*	6.9*10^−3^	0.083
Total lysoPC	*−0.1369*	0.020	0.073
CPT‐I ratio^d^	*−0.1328*	2.7*10^−3*^	0.042
PC ae C36:5	0.1758	2.2*10^−4*^	3.8*10^−3*^
PC ae C36:4	0.1722	1.5*10^−3*^	4.9*10^−3*^
PC ae C38:6	0.1491	7.9*10^−5*^	0.018
PC ae C38:5	0.1427	0.017	0.023
Alpha‐AAA	0.1418	0.060	0.059

^a^ Negative *loadings* indicate lower concentration in natural bodybuilders. Positive loadings indicate higher concentration in natural bodybuilders compared to all other groups.

^b^ PC ae C38:0 is isobar (same nominal mass) with PC aa C38:7. In human plasma of young healthy men, PC ae C38:0 is considered to contain considerable amounts of PC molecules that carry a fatty acid chain with 22 carbon atoms and 6 double bonds (C22:6), same as for the related measure PC aa C36:6 (Quell et al., 2019). SM (OH) C22:1 and SM (OH) C22:2 labeled as hydroxy‐sphingolipids are isobar with odd‐chain non‐hydroxy sphingolipids (e.g. SM C23:0 and SM C23:1).

^c^ *Negative loadings* indicate higher concentration in endurance athletes. Positive loadings indicate lower concentration in endurance athletes compared to all other groups.

^d^ Sum of hexadecanoylcarnitine (C16:0) and octadecanoylcarnitine (C18:0) divided by free carnitine (C0).

* Significant comparison after correcting for multiple testing *p* < 5.0*10^−3^.

**FIGURE 3:**
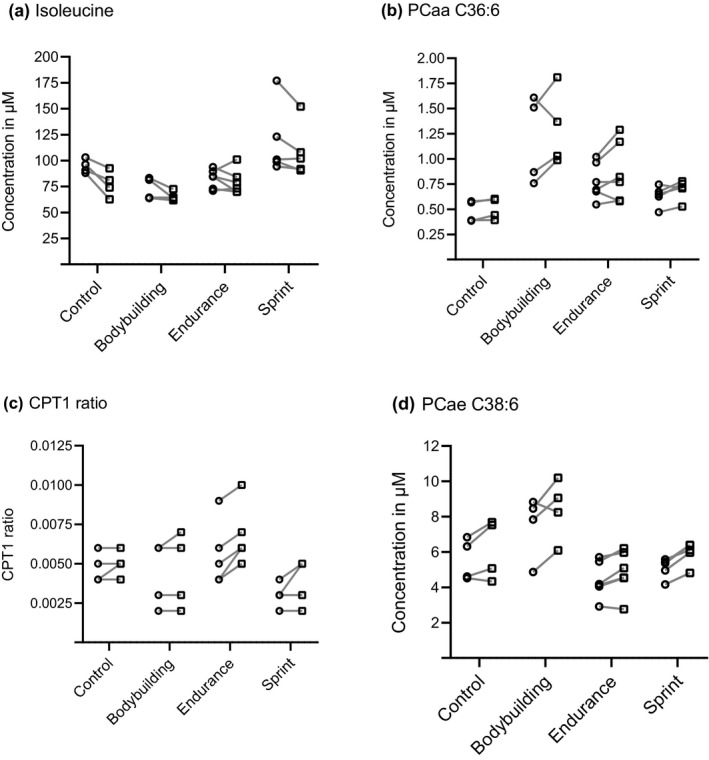
Concentration changes for every participant between *baseline* (○) and *post*‐*exercise* (□) for isoleucine (a) and PC aa C36:6 (b), contributing most to the separation of natural bodybuilders and the CPT1‐ratio (c) and PC ae C38:6 (d) contributing most to the separation of endurance athletes

Isoleucine, leucine, BCAA, tryptophan and tetradecadienoylcarnitine (C14:2) were lower concentrated in natural bodybuilders (Figure 3a) when compared to all other groups. Five complex lipids including two hydroxy‐sphingolipids (SM‐OH), the total sum of hydroxy‐sphingolipids (total SM(OH)) and two PCs (Table 2a) were higher concentrated in natural bodybuilders (Figure 3b) when compared to all others. Out of the 10 metabolites selected in component 1, PC aa C36:6, PC ae C38:0, and C14:2 differed significantly (*p* < 5.0*10^−3^) between bodybuilders and all other groups when tested one by one (Table 2a).

Alpha‐aminoadipic acid (alpha‐AAA) and four PCaes (Table 2b, Figure 3d) were lower concentrated in the endurance athletes when compared to all other groups. In contrast, three lysoPC measures, the kynurenine/tryptophan ratio and the CPT1‐ratio (Figure 3c) which is a proxy measure for a rate‐limiting step in ß‐oxidation, were higher concentrated in endurance athletes than in all other groups (Table 2b). Out of the 10 metabolites selected in component 2, PC ae C36:4 and PC ae C36:5 differed significantly (*p* < 5.0*10^−3^) between endurance athletes and all groups when tested one by one (Table 2b).

### 3.3 How does a fasted, graded exercise test to exhaustion affect blood metabolite concentrations?

**FIGURE 4:**
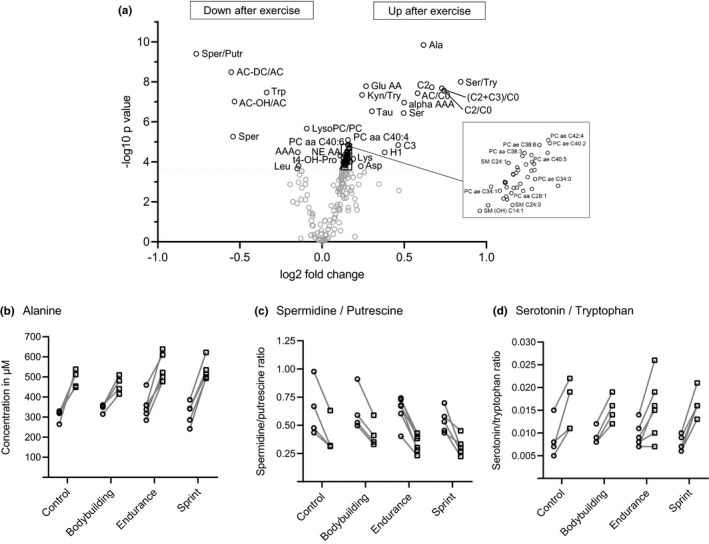
Volcano plot (a) showing significant metabolite changes (in black; α < 2.58*10^−4^) after graded cycle exercise in all participants and metabolites with the highest concentration changes from baseline (○) to post‐exercise (□) including (b) alanine, (c) spermidine/putrescine and (d) serotonin/tryptophan

Next, we compared the concentrations of 151 metabolites and 43 metabolite ratios or sums between post‐exercise and baseline. After exercise, a third of all metabolite measures (46 metabolites and 12 metabolite ratios or sums) significantly increased. In contrast, only ~5% of all metabolite measures (4 metabolites, 5 ratios or sums) significantly decreased after exercise (Figure 4, Supplementary Table S5).

In detail, the ratio spermidine/putrescine decreased most, while the ratio serotonin/tryptophan increased most across groups (tryptophan decreasing; serotonin increasing). Alanine concentration increased and had the lowest *p*‐value (Figure 4a) of metabolites that changed by exercise. Among all amino acids, muscles mainly excrete alanine during fasting, and the blood transports it to the liver for gluconeogenesis (Adeva‐Andany et al., 2016). After exercise, the ratio of short chain acylcarnitines to free carnitine (C2+C3/C0), the ratio of acetylcarnitine to free carnitine (C2/C0), short chain acylcarnitines (C2, C3) and the ratio of esterified to free carnitine (total AC/C0) increased, indicating higher ß‐oxidation activity. Several complex lipids like PCs and SMs increased after exercise (Figure 4a), suggesting a general increase in blood complex lipids after exercise.

### 3.4 Do metabolite concentrations change differently after exercise depending on the group?

Finally, we compared the log2 fold‐change of each metabolite between groups (Supplementary Table S6). No metabolite change differed significantly between groups when correcting for 194 tests (151 metabolites, 43 ratios or sums). However, when using a raw *p*‐value cut‐off of *p* < 0.01, we identified metabolites with suggestive, group‐specific responses to exercise.

**FIGURE 5:**
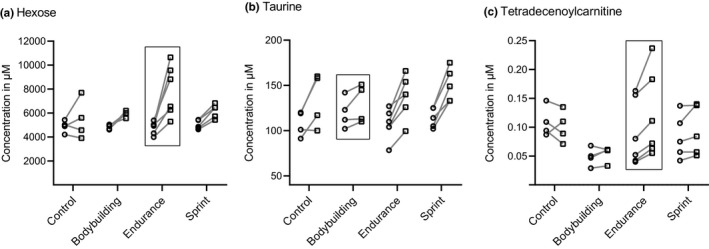
Among all 194 metabolite measures, hexose (a), taurine (b) and tetradecenoylcarnitine (c) showed suggestive group‐specific responses between baseline (○) and post‐exercise (□)

In endurance athletes, hexose (Figure 5a), which mainly comprises glucose (fasting blood glucose concentration in healthy humans ranges between 4.0 and 5.9 mmol/l) (American Diabetes Association, 2014), butyrylcarnitine (C4), tetradecenoylcarnitine (C14:1), and tetradecadienoylcarnitine (C14:2), had higher fold‐changes in response to exercise compared to all other groups. In natural bodybuilders, putrescine and taurine (Figure 5b) stayed almost at the same level form pre‐to post exercise, whereas in all other groups, putrescine and taurine increased. In the control group, C14:1 and tetradecanoylcarnitine (C14:0) had lower fold‐changes compared to all other groups. Notably, C14:1 was one of those metabolites which increased the highest in endurance athletes.

In sprinters, we found no metabolite that had a *p*‐value below 0.01. The metabolites that differed most were serotonin (*p* = 0.029) and the serotonin/tryptophan ratio (Figure 4d) with higher, but not significant, increases in sprinters when compared to all other groups.

## 4 DISCUSSION

The aim of this study was to investigate how the selectively adapted metabolism of aerobic, glycolytic, and anabolic athletes affects blood metabolomes at rest or after exercise and in response to exercise. We made the following main observations: First, endurance‐trained athletes and natural bodybuilders had unique metabolite concentrations, ratios and sums when compared to sprinters and untrained controls. Second, endurance athletes had higher CPT1‐ratios, higher lysoPCs C18:1 and C18:2 and lower levels of highly unsaturated alkyl‐acyl‐phosphatidylcholines than others. In contrast, natural bodybuilders had lower concentrations of BCAAs, lower tryptophan and higher concentrations of specific phosphatidylcholines and sphingomyelins. Third, ~30% of all serum metabolite measures increased 5 minutes after a graded bicycle ergometry test to exhaustion, whereas ~5% of all metabolite measures decreased. Fourth, some metabolites changed differently during exercise in‐between groups but not significantly.

The first and the second main findings are discussed together, followed by the third and the fourth finding separately.

### 4.1 Natural bodybuilders have a depleted blood BCAA pool, which might be caused by high muscle growth

Fasted, natural bodybuilders had lower concentrations of leucine, isoleucine, tryptophan, and BCAAs than the other groups. A likely explanation is that higher rates of protein synthesis (McGlory et al., 2017) and a greater muscle mass result in faster declines of amino acids including BCCAs. The standardized nutrition on the day before testing may have contributed to this finding: Based on their dietary reports, natural bodybuilders usually ingested higher amounts of protein (~36%, ~2.4 g/kg bodyweight) than all other groups (20–24%, ~0.9–2.0 g/kg bodyweight). On the day before testing, protein intake was standardized to 20% of total macronutrient intake for all participants. For bodybuilders, this reduced protein intake could have depleted BCAA and tryptophan in blood even faster because bodybuilders need more of these dietary essential amino acids than the other groups due to higher protein synthesis rates. A practical conclusion to the fast overnight depletion of blood amino acids could be that bodybuilders should consider ingesting protein pre‐and post‐sleep to avoid “running empty” on amino acids.

In general, habitual dietary protein intake can also influence amino acid levels in blood (Durainayagam et al., 2019; Seyedsadjadi et al., 2018). Durainayagam et al. report that doubling protein intake (from 0.8 g/kg bodyweight to 1.6 g/kg bodyweight) for 10 weeks increases tryptophan, creatine, and glutamine levels. Seyedsadjadi et al. report that the intake of ~94 g protein per day increases tryptophan and kynurenine but do not provide data on relative protein intake in g/kg bodyweight. Contrasting to both studies, tryptophan levels in bodybuilders were lower than in all other groups in our study. We are not aware of any study that has shown how the habitually high protein intake of the participating natural bodybuilders (~36%) affects the blood metabolome long‐term.

### 4.2 Natural bodybuilders have higher levels of two docosahexaenoic acid derivatives which may originate from supplemented fish oils

PC aa C36:6 and PC ae C38:0 concentrations were higher in natural bodybuilders when compared to all other groups, with the largest differences observed for PC aa C36:6. Quell et al. recently showed that blood PC aa C36:6 measured in healthy young men mainly comprises a derivative of DHA, a fatty acid with 22 carbons and 6 double bonds (C22:6). Quell et al. found PCaas that contain DHA as one, and tetradecanoic acid as the second fatty acid chain (PC 14:0_22:6) account for the major part (~88%) of measured PC aa C36:6 concentrations (Quell et al., 2019). They also suggested the second PCae that was higher in natural bodybuilders (PC ae C38:0) to be a DHA derivative. The measure labeled as PC ae C38:0 includes the concentrations of the isobaric (i.e., showing the same mass spectrometric signal) PC aa C38:7 with C22:6 as one of the two fatty acid chains. Natural bodybuilders may have higher levels of DHA‐containing PCs as some bodybuilders supplemented fish oils. Fish oils are rich in omega‐3 fatty acids including DHA and eicosapentaenoic acid (EPA, C20:5) and have been reported to increase muscle protein synthesis via increased mTOR and p70 S6 k signaling (Smith et al., 2011). Especially two natural bodybuilders (B3 and B4) who either ingested omega‐3 capsules (B3, Supplementary table S3) or ate omega‐3 rich oils (around 20 g daily), chia seeds (around 20 g daily), and fish (weekly) (B4) had high concentrations of DHA‐containing PCs. Among the other participants, only endurance athlete E3 ate fish regularly. E3 had the highest baseline concentration in PC aa C36:6 next to B3 and B4, but no notable elevation in PC ae C38:0. No other participant reported rapeseed oil, linseed oil, or chia seeds or fish in their nutrition. Besides the DHA derivatives, the concentrations of two sphingomyelins which also contain fatty acids with 22 carbons were higher in natural bodybuilders than in all other groups. Whether this is similarly a result of the natural body builders diet or because other factors play a role is unclear. Collectively, the overall pool of 22 carbon fatty acid‐containing molecules such as PCs or sphingomyelins is higher in natural bodybuilders, which might be in part because of their diets.

### 4.3 Endurance athletes have higher CPT1‐ratio, suggesting higher fat oxidation

Endurance athletes had higher CPT1‐ratios than all others, especially sprinters. CPT1 is a mitochondrial transmembrane enzyme that catalyses a reaction essential for the transport of fatty acids from the cytosol into the mitochondria, where fatty acids enter β‐oxidation (Lundsgaard et al., 2018). Metabolomics analyses allow to estimate the activity of the CPT1 reaction via its reaction products hexadecanoylcarnitine (C16:0) and octadecanoylcarnitine (C18:0) versus the concentration of free carnitine (C0). The CPT1‐ratio could be a biomarker either for a higher capacity for fat oxidation or for acutely higher fat oxidation rates in endurance athletes. Supporting this assumption, endurance athletes had the lowest respiratory exchange ratios (RERs) of all groups, at rest and during the exercise test, indicating higher fat oxidation compared to the other groups (Supplementary Figure S3). Further studies should seek to clarify the association between the CPT1‐ratio, the capacity for fat oxidation, CPT1 activity and the acute rate of fat oxidation.

### 4.4 Endurance athletes have higher lysoPC a C18:1 and C18:2 concentrations, which may be linked to cardiovascular fitness

Endurance athletes had higher concentration of lysoPC a C18:1 than bodybuilders and controls. Earlier studies have already associated lysoPCs containing 18 carbons with endurance traits (Felder et al., 2017; Schader et al., 2020). Specifically, lysoPC a C18:1 was, among other lysoPCs, reported to increase after several weeks of endurance training (Felder et al., 2017). Another lysoPC C18:2 was found to be elevated after a marathon race in subjects with high VO_2_max (63.3 ± 5.2 ml/kg/min) that is similar to the VO_2_max of our endurance athletes (Table 1), but not in subjects with low VO_2_max (41.8 ± 5.5 ml/kg/min) (Schader et al., 2020) that is similar to the VO_2_max of natural bodybuilders and controls (Table 1). Supporting the association between cardiovascular fitness and these lysoPCs, lysoPC a C18:0 and lysoPC a C18:2, were shown to be lower in patients with heart failure, who typically have a lower VO_2_max (17.2 ± 7.2 ml/kg/min), than in healthy controls (Marcinkiewicz‐Siemion et al., 2018). LysoPCs are generated by phospholipases A (PLA) from PCs. Overexpression of a specific PLA, phospholipase A2 type IIA (PLA2G2A), which is secreted to blood and expressed in skeletal muscle and adipose tissue (Prunonosa Cervera et al., 2021), increased the metabolic rate, and improved both insulin sensitivity and glucose tolerance in mice (Kuefner et al., 2017). Interestingly, mice expressing PLA2G2A compared to mice without PLA2G2A expression had higher uncoupling protein 1 (UCP‐1) and higher peroxisome proliferator‐activated receptor‐gamma coactivator‐1‐alpha (PGC1‐alpha) expression in adipose tissue, suggesting a role of PLA2G2A in adipose tissue browning (Kuefner et al., 2017). Among others PGC1‐alpha is known as a major regulator for mitochondrial biogenesis after endurance exercise in humans. Furthermore, there is first evidence in humans that exercise can increase PLA2G2A expression in adipose tissue (Imam, 2019). Collectively, increased lysoPCs in endurance athletes may point to exercise‐associated increases in specific phospholipases that are beneficial for metabolic health.

### 4.5 Endurance athletes have lower levels of highly unsaturated PCaes, which are ligands of endurance adaptation regulators

Endurance athletes had lower concentrations of specific PCaes (PC ae C36:5, PC ae C36:4, PC ae C38:6) than all other groups. Functionally, PCae can act as ligands of signaling molecules like PPARγ (Dean & Lodhi, 2018), which is a known regulator of the mitochondrial biogenesis adaptation to endurance exercise. We therefore speculate that endurance exercise decreases certain blood PCaes, because they are needed in intramuscular signaling for signaling processes in adaptation.

### 4.6 In all subjects, fasted, graded cycle ergometry to exhaustion affects energy metabolism

Consistent with other studies (Contrepois et al., 2020; Morville et al., 2020; Schranner et al., 2020), most metabolites that changed after exercise in all participants were energy metabolites related to glucose or fat degradation. Specifically, the concentrations of gluconeogenic and glycolytic metabolites such as alanine and hexose (mainly glucose) increased after exercise. Increases in blood glucose concentration during exercise probably result from hepatic glucose production via glycogenolysis and gluconeogenesis (Brooks, 2020; Kjær, 1998). Measures of overall fatty acid oxidation activity (C2+C3/C0), even‐numbered fatty acid oxidation activity (C2/C0) and the concentrations of short chain acylcarnitines (C2, C3) all increased. Exercise is known to increase lipolysis in fat tissue and fat oxidation within mitochondria (Lundsgaard et al., 2018), leading to increased acylcarnitine levels in blood (Schranner et al., 2020).

### 4.7 In all subjects, exercise increases tryptophan breakdown to serotonin and kynurenine, which links to mental health effects of exercise

Exercise increased the ratios of serotonin/tryptophan and kynurenine/tryptophan as well as increased the serotonin concentration and decreased the tryptophan concentration. In contrast, kynurenine concentrations did not change significantly. Thus, we assume that the increase in kynurenine/tryptophan is only because tryptophan decreased, suggesting that exercise shifted the tryptophan breakdown towards serotonin. Serotonin can be broken down to melatonin and positively regulate mood or sleep (De Crescenzo et al., 2017; Zimmer et al., 2016). Serotonin increases were shown to depend on exercise intensity (Zimmer et al., 2016), which explains the significant increase of serotonin after maximum exercise done in our study. Supporting our findings, Strasser et al. also found decreased tryptophan concentrations in athletes after exercise (Strasser et al., 2016). Collectively our data confirm that acute exercise alters metabolites that are associated with mood and mental health.

### 4.8 In all subjects, exercise increases complex lipids, especially PCaes

Complex lipids can act as ligands for cell signaling and can be used for fat oxidation. We assume that increased concentrations of complex lipids after maximum exercise are a sign of increased lipolysis and oxidation in the fasted state. In line with that, total PCae concentrations increased in rat livers after acute exercise (Hoene et al., 2016), which could show a higher demand for muscular fatty acid oxidation. In contrast, several studies reported that complex lipids decrease after exercise in non‐fasted humans (Karl et al., 2017; Nieman et al., 2013; Schader et al., 2020). Therefore, we assume that in fasted but not in non‐fasted subjects, complex lipids like PCae or PCaa are used for fat oxidation during exercise.

### 4.9 In all subjects, exercise increases polyamines, which are related to muscular hypertrophy

Spermidine and the spermidine to putrescine ratio decreased after exercise. As putrescine did not change, the observed decrease in the spermidine to putrescine ratio is mainly because of the decrease in spermidine. Polyamine concentrations in skeletal muscle are associated with hypertrophy (Cepero et al., 1998; Turchanowa et al., 2000) and muscle regeneration after injury (Kaminska et al., 1982) in rats. We assume that the blood spermidine pool decreases after exercise not because of changed spermidine synthesis, but because of higher spermidine demand in muscle after exercise e.g. to regenerate. Mechanistically, it is still unclear why muscular polyamine concentrations increase after exercise (Lee & MacLean, 2011) but it seems that androgens like testosterone, which also increase muscle mass and strength, regulate polyamine synthesis (Cyriac et al., 2002). Eventually, it is unclear if and how polyamine concentration changes after exercise in blood relate to intra‐muscular processes.

### 4.10 In endurance athletes, blood glucose concentration increased more after exercise than in all other groups

Despite the low sample size and lack of significance after stringent multiple testing correction, we briefly discuss metabolites that changed differently in‐between groups after exercise. Specifically, we highlight the differences found in endurance athletes.

After exercise, hexose (mainly glucose) concentrations increased in all athletes but most in endurance athletes. Despite strenuous exercise, hexose increased only in 2 out of 4 untrained controls. Intensive exercise increases hepatic glucose production via glycogenolysis and gluconeogenesis by 2–3 fold (Brooks, 2020) to prevent hypoglycemia. Collectively, this suggests that endurance athletes either have a high ability for hepatic glucose synthesis and/or less muscular glucose uptake because of higher rates of fat oxidation during submaximal exercise.

### 4.11 In endurance athletes, medium/long‐chain acylcarnitines increased more after exercise than in all other groups

During exercise, the concentrations of C14:1 and C14:2, which are involved in the ß‐oxidation of long‐chain fatty acids, increased highest in endurance athletes especially when compared to untrained controls. We assume that this shows different usage or availability of long‐chain fatty acids for fat oxidation. Recently, several other long‐chain acylcarnitines have been associated with endurance exercise variables (Al‐Khelaifi et al., 2018). Collectively, this suggests that endurance athletes may metabolize long‐chain fatty acids differently than subjects who are not endurance trained.

### 4.12 Limitations

This study has several limitations. First our study cohort was small, restricting the statistical power for group comparisons. We justify this small cohort with the large differences of glycolytic capacity, aerobic capacity, and anabolism in the four groups investigated. Following a hypothesis‐free approach, we indeed observed significant differences in the metabolomes of these highly selective groups, despite the relatively low sample size and variations within groups. However, due to the limited statistical power in our study and because results of the PLS‐DA might be biased towards the groups with the biggest differences observed (bodybuilders vs. all others and endurance vs. all others), we might have missed less pronounced differences, in particular potential differences between sprinters and controls. Therefore, we cannot draw any robust conclusion on the differences between these two groups. Still, we consider that the metabolomes of sprinters and controls are more similar than those of the other groups in this study. Moreover, the choice of PLS‐DA might have biased the selection of metabolites for differential analysis (Ruiz‐Perez et al., 2020). Second, we only used endurance exercise as an exercise mode, which activates only a subset of metabolic enzymes in the musculature. Other exercise modes such as resistance exercise would have stimulated other branches of metabolism (Morville et al., 2020) and may have revealed other group‐specific changes in the metabolome. With choosing a standardized maximum endurance test, we did not aim to report metabolite changes of endurance training per se but those of a metabolic challenge to metabolism. Contrepois et al. showed that such standardized maximum exercise testing is sufficient to show phenotypic differences in metabolism (Contrepois et al., 2020). Third, we measured a limited set of metabolite classes mainly lipids (e.g. acylcarnitines) and amino acids. We assumed that acute, fasted exercise particularly challenges lipid energy metabolism and shows differences between athlete groups with differently well‐developed lipid metabolism. Furthermore, we assumed that especially natural bodybuilders, who have high protein synthesis, have different baseline amino acid levels than other athletes. Amino acids were also of interest because previous studies inconsistently reported amino acid changes after exercise (Schranner et al., 2020). Fourth, after study completion, subject E5 reported a nightly ingestion of ~1 mg of melatonin, which was against our inclusion criteria. Studies suggest that melatonin has effects on several organs (Opie & Lecour, 2016) besides the brain. Possibly, this influenced E5’s metabolite levels at baseline, post‐exercise or the level changes by exercise. Specifically, melatonin ingestion can affect metabolites of its related pathway, the tryptophan‐serotonin pathway. In the PLS‐DA, E5 appeared metabolically different from the cluster of E1‐E4. However, this difference is not attributable to different serotonin levels, as E5 showed no conspicuous serotonin levels compared to E1‐E4. Fifth, special nutrition or dietary supplements may influence certain metabolite concentrations long‐term. As suggested by higher DHA levels in natural bodybuilders in our study, refraining from dietary supplements for 48 hours before a metabolomics analysis may not be long enough to eliminate all nutritional influences on certain blood metabolites. However, it is also questionable if longer restriction of supplements for several days is enough to wash out long‐term dietary influences. Studies that investigated dietary effects on the metabolome assessed diets between 2 weeks and 6 months (Guasch‐Ferré et al., 2018). Controlling supplementary intake that long is problematic when working with ambitious athletes. Sixth, we additionally provided 500–1000 kilocalories for athletes (Supplementary Table S7) on the day before the study as they have higher energy demand than sedentary controls. Increased caloric intake included all classes of macronutrients but slightly higher fat intake (~25%) when compared to controls (~20%). Dietary fat intake on the day before the study could have influenced acylcarnitine levels of the natural bodybuilders because they habitually ate low‐fat (~14.7%). Low levels of C14:2 acylcarnitine have been associated with higher intake of fats such as butter (Floegel et al., 2013). Complex lipids like PCae's and PCaa's are not influenced by short term but by long‐term fat intake over weeks and months (Saadatian‐Elahi et al., 2004). Seventh, as expected in highly specialized athletes, we observed significant differences in body fat and suggestive differences in muscularity between groups (Table 1). A population‐based study by Jourdan et al., (2012) found that a high fat free mass index (FFM kg/height²) which corresponds to low body fat, was associated with higher levels of BCAAs, acylcarnitines, and a shift in phosphatidylcholine composition, chain length and saturation (Jourdan et al., 2012). In the case of BCAA, we see the highest concentrations in the group with the lowest body fat (sprinters) but no consistent association, across our cohort.

## 5 CONCLUSION

In conclusion, we found systematic differences in the concentrations of metabolites in‐between highly trained glycolytic, aerobic, and anabolic athletes. Moreover, we observed different metabolite changes in‐between groups that were not significant but worth of mentioning. The observed metabolic differences of years of training could give hints on which exercise mode can change specific metabolites or metabolite classes. However, influences on the metabolome are manifold and further studies are needed to disentangle the specific contributions of genetic variants, of adaptations to sports‐specific exercise training or of special nutrition to the systematic metabolic differences between differently trained individuals.

## CONFLICT OF INTEREST

The authors declare that they have no competing financial or non‐financial interests.

## AUTHOR CONTRIBUTIONS

HW had the idea for the study. HW and MS designed the study. DS, MS, JS (Johannes Scherr), JS (Jürgen Schlegel), OZ, AR, SK, MH, QS, FS, and FK conducted human experiments and gathered the data. CP and JA measured the samples. DS, WRM, and GK analyzed the data. DS, MS, GK and, HW interpreted the results and wrote the manuscript. All authors proof‐read and approved the final version of the manuscript.

## ETHICAL APPROVAL STATEMENT

This study conforms to the Declaration of Helsinki for use of human subjects and tissue and was approved by the medical ethics committee of the Technical University of Munich (356/17S).

## Consent to participate

Participants were fully informed of the nature and possible risks of the study before they gave their written informed consent.

## Consent for publication

Participants were fully informed that their data will be made publicly available to the scientific community after anonymization.

## Supporting information



Supplementary Material

## Data Availability

The MS raw data and sample information of this study will be made available within the MetaboLights (Haug et al., 2020) repository under the accession number MTBLS2104 (https://www.ebi.ac.uk/metabolights/MTBLS2104).
